# Acute cardiogenic dyspnea in the emergency department: accuracy of lung ultrasound

**DOI:** 10.1186/2036-7902-6-S2-A7

**Published:** 2014-08-27

**Authors:** A Spinelli, E De Curtis, A Savino, G Michelagnoli, S Magazzini

**Affiliations:** 1 Department of Emergency Medicine, S. Stefano Hospital, Prato, Italy; 2 Department of Critical Care, S. Stefano Hospital, Prato, Italy

## Background

Acute dyspnea is a common symptom in the emergency department and several studies have investigated the usefulness of Lung Ultrasound (LUS) in the approach to the patient with dyspnea with different results.

## Objective

On the basis of the literature, we performed a prospective observational study, aiming to assess the accuracy of LUS for the diagnosis of cardiogenic dyspnea in patients presenting at the emergency department with acute dyspnea.

## Patients and methods

70 patients presenting with acute dyspnea have been enrolled.

On admission every patient underwent to LUS and chest X-ray (CXR). Ultrasound findings of Interstitial Alveolar Syndrome (IAS), which correlate with an increase in interstitial lung water, were reported. On the results of LUS, the patients were attributed to 2 different diagnostic groups: Group 1: patients with cardiogenic dyspnea, Group 2: patients with respiratory dyspnea. CXR was performed as gold standard exam to discriminate the respiratory and cardiogenic origin of the dyspnea. At the discharge from the hospital, two investigators blind to the results of the LUS exam, revised all the clinical charts and attributedevery patient to a definitive diagnostic group. On the basis of the results, it was possible to calculate Sensitivity, Specificity, Positive Predictive Value, Negative Predictive Value of LUS and CXR for the diagnosis of cardiogenic dyspnea.

## Results

LUS showed a sensitivity of 92%, Specificity 93%, PPV 88%, NPV 95%; while CXR showed Sensitivity 79%, Specificity 74%, PPV 61%, NPV 87%. Differences between Specificity and PPV were significant (Chi-Square test, P<0.05).

## Conclusion

LUS can be more effective than CXR for the diagnosis of cardiogenic dyspnea.
